# More Than Muscle Aches: A Case of Statin-Associated Necrotizing Myositis

**DOI:** 10.7759/cureus.113089

**Published:** 2026-07-21

**Authors:** Angela Chen, Laure Deliscar, Kylie Ditty, Anthony Moon

**Affiliations:** 1 Osteopathic Medicine, Nova Southeastern University Dr. Kiran C. Patel College of Osteopathic Medicine, Clearwater, USA; 2 Medicine, Nova Southeastern University Dr. Kiran C. Patel College of Osteopathic Medicine, Clearwater, USA; 3 Osteopathic Medicine, Nova Southeastern University Dr. Kiran C. Patel College of Osteopathic Medicine, Fort Lauderdale, USA; 4 Family Medicine, Lakeland Regional Health, Lakeland, USA

**Keywords:** anti-hmg-coa reductase antibody, immune-mediated necrotizing myopathy, intravenous immunoglobulin, statin myopathy, transaminitis

## Abstract

Statin-associated immune-mediated necrotizing myopathy (SINAM) is a rare but increasingly recognized autoimmune condition in which progressive proximal muscle weakness develops in statin-exposed patients and persists after drug discontinuation, driven by autoantibodies targeting HMG-CoA reductase. We report a 70-year-old woman who developed subacute progressive bilateral lower-extremity weakness on a background of nearly a year of asymptomatic, gradually worsening elevation of aspartate aminotransferase (AST) and alanine aminotransferase (ALT), the standard liver enzymes, following more than three years of atorvastatin therapy. Diagnosis of anti-HMG-CoA reductase (anti-HMGCR) antibody-positive immune-mediated necrotizing myopathy (IMNM) was established by an isolated anti-HMGCR antibody level of 209 units (normal less than 20 units) in the setting of a uniformly negative standard myositis-specific antibody panel, confirming that anti-HMGCR must be requested as a separate assay. She was managed with atorvastatin discontinuation, high-dose corticosteroids, and escalation to intravenous immunoglobulin (IVIG) following biochemical relapse on steroid taper. This case highlights three teaching points: skeletal muscle as a frequently overlooked source of AST and ALT elevation in statin-treated patients, the necessity of dedicated anti-HMGCR testing when persistent post-statin myopathy is suspected, and the emerging serologic subtype-specific approach to second-line immunotherapy in IMNM.

## Introduction

Anti-HMG-CoA reductase (anti-HMGCR) antibody-positive immune-mediated necrotizing myopathy (IMNM) is a serologically defined subset of the inflammatory myopathies that is increasingly recognized in patients with current or remote statin exposure [1-3]. Historical incidence estimates of one to two per 100,000 statin users per year have been supplanted by more recent data, including a 2026 single-center Finnish cohort reporting 2.75 per 100,000 statin users per year [4]. A large Australian population-based case-control study demonstrated an approximately two-fold increased likelihood of statin exposure among patients with histologically confirmed idiopathic inflammatory myositis, with an adjusted odds ratio of 1.79 and a 95% confidence interval of 1.23 to 2.60 [5].

Diagnosis is often delayed due to a convergence of factors: the relative rarity and evolving clinical recognition of the disease; significant clinical overlap with other inflammatory myopathies, Guillain-Barre syndrome, and structural causes of weakness; the tendency to attribute early transaminase elevations to hepatocellular injury rather than skeletal muscle in statin-treated patients; and the fact that anti-HMGCR antibody is not included in standard myositis-specific autoantibody panels and must be requested as a separate assay [3,6]. The American Heart Association (AHA) has published guidance distinguishing hepatic from muscle sources of transaminase elevation in statin-treated patients, which is directly relevant to the diagnostic delay illustrated by this case [2]. We describe a case in which several of these contributing factors played out simultaneously over nearly a year of asymptomatic biochemical evolution before overt weakness prompted hospitalization, underscoring the importance of measuring creatine kinase (CK) when transaminase elevations occur in a statin-treated patient.

## Case presentation

**Figure 1:**
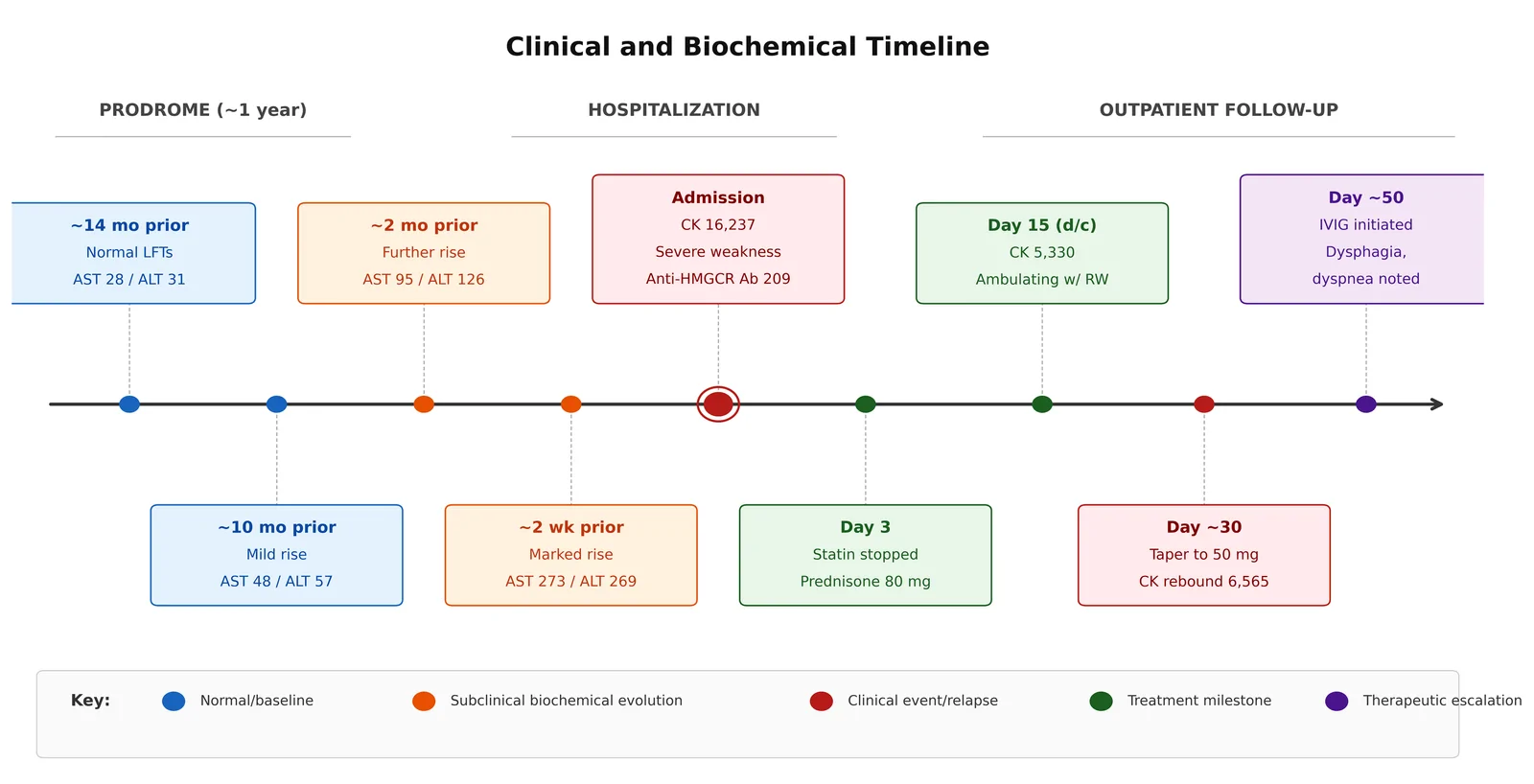
Clinical and biochemical timeline. LFTs = liver function tests; CK = creatine kinase; Ab = antibody; RW = rolling walker; d/c = discharge. Time axis is not to scale; intervals are approximate.

A 70-year-old woman with a past medical history of hypertension and dyslipidemia presented with a four-to-six-week history of progressive bilateral lower-extremity weakness, edema, and paresthesias. The clinical and biochemical timeline of her presentation, hospitalization, and outpatient follow-up is illustrated in Figure 1.

Pre-morbid laboratory trajectory

In retrospect, the patient's laboratory data demonstrated a gradual, asymptomatic biochemical evolution that preceded her motor symptoms by nearly a year. Routine outpatient chemistries approximately 14 months prior to admission were unremarkable, with AST 28 U/L (normal range 10-35 U/L) and ALT 31 U/L (normal range 10-35 U/L). Approximately 10 months prior to admission, mild transaminase elevations had emerged (AST 48 U/L, ALT 57 U/L) without symptoms. Surveillance laboratories approximately two months prior to admission revealed further elevation (AST 95 U/L, ALT 126 U/L), and a panel obtained approximately two weeks before admission demonstrated AST 273 U/L, ALT 269 U/L, total bilirubin 1.1 mg/dL (normal range 0.2-1.2 mg/dL), and an erythrocyte sedimentation rate of 31 mm/hr (normal range 0-30 mm/hr for women over 50). At each of these intervals, the transaminase elevation was attributed to possible statin-induced hepatotoxicity; hepatic imaging was not performed at that stage, and the statin was not yet discontinued. The significance of these findings as a marker of subclinical skeletal muscle injury was not recognized until after admission.

Notably, ALT exceeded AST in both intermediate prodromal specimens, a pattern more consistent with hepatocellular than muscle injury under the framework outlined in the AHA scientific statement on statin safety [2], and the ratio shifted toward parity (AST and ALT nearly equal) only on admission. Per pharmacy records, atorvastatin had been initiated approximately 40 months prior to admission. The patient therefore tolerated statin therapy without clinical or laboratory abnormality for approximately the first two years of exposure, with mild transaminase elevation first detectable approximately 10 months prior to admission and progressive rise thereafter.

Initial clinical presentation

In the weeks preceding admission, the patient developed bilateral lower-extremity edema with a sensation of heaviness, "like dragging lead," most prominent in the posterior thighs and calves, accompanied by tingling and numbness predominantly on the left. Symptoms were constant, unrelieved by rest, and not provoked by exertion. She reported difficulty rising from a seated position and inability to lift her legs while supine. Prior to symptom onset, she had been independent in all activities of daily living and ambulated one to two hours daily, including participation in group exercise classes. Her home medications included atorvastatin (maintained for over three years without prior intolerance), semaglutide administered by subcutaneous injection in the lower extremities, and losartan for hypertension. She initially attributed her symptoms to the semaglutide injection site.

Hospital course

The patient was admitted to a regional hospital for further evaluation. Admission laboratory findings, including units of measurement and normal reference ranges, are summarized in Table 1.

**Table 1:** Summary of admission laboratory findings. *Exact numeric value not available in source records; flagged as critical by the reporting laboratory.

Test	Result	Units	Normal range
Creatine kinase (CK)	16,237	U/L	26-192
Aspartate aminotransferase (AST)	388	U/L	10-35
Alanine aminotransferase (ALT)	407	U/L	10-35
Total bilirubin	1.4	mg/dL	0.2-1.2
Direct bilirubin	0.5	mg/dL	less than 0.3
High-sensitivity troponin-T	Critically elevated*	--	--
Erythrocyte sedimentation rate (ESR)	31	mm/hr	0-30 (women over 50)
C-reactive protein (CRP)	4.7	mg/L	less than 3.0
Anti-HMG-CoA reductase (anti-HMGCR) antibody	209	units	less than 20
Myositis-specific antibody panel (Jo-1, OJ, PL-7, PL-12, EJ, SRP, MDA5, Mi-2α, Mi-2β, NXP-2, TIF1-γ)	Negative, all <11	SI units	less than 11
Hepatitis A IgM	Negative	--	--
Hepatitis B surface antigen	Negative	--	--
Hepatitis B core IgM	Negative	--	--
Hepatitis C antibody	Positive	--	--
Hepatitis C virus RNA (PCR)	Not detected	--	--
Cerebrospinal fluid protein	22.9	mg/dL	15-45
Cerebrospinal fluid nucleated cell count	1	cells/µL	0-5
Urinalysis: leukocyte esterase	2+	--	Negative
Urinalysis: protein	1+	--	Negative
Urinalysis: blood	2+	--	Negative

At the time of admission, the principal differential diagnoses included: (1) inflammatory myopathy or immune-mediated necrotizing myopathy, given the subacute proximal weakness and markedly elevated CK; (2) Guillain-Barre syndrome, given the progressive bilateral limb weakness, diminished deep tendon reflexes, and ascending pattern; (3) acute ischemic stroke or intracranial process, given the acute presentation and the need to exclude central nervous system pathology before attributing weakness to a peripheral or muscular cause; and (4) cervical myelopathy, given multilevel spondylosis on prior plain films. Non-contrast CT of the head and gadolinium-enhanced MRI of the brain were obtained to systematically exclude acute intracranial hemorrhage and ischemic stroke, which are essential diagnostic considerations in any patient presenting with new progressive limb weakness of unclear localization, regardless of the eventual diagnosis. These studies, along with MRI of the cervical, thoracic, and lumbar spine, identified only incidental degenerative spondylosis and a T5 vertebral hemangioma, excluding central and compressive structural etiologies.

A neurology consultant evaluated the patient on hospital day 3 and recommended lumbar puncture based on the clinical features most consistent with Guillain-Barre syndrome at that juncture: subacute progressive bilateral lower-extremity weakness with a proximal-to-distal gradient, globally diminished or absent deep tendon reflexes, and the absence of a confirmed alternative diagnosis. Although the Brighton Collaboration criteria for Guillain-Barre syndrome do not require cerebrospinal fluid analysis for diagnosis, lumbar puncture was performed to evaluate for the characteristic albuminocytologic dissociation, which, if present, would have supported early initiation of plasma exchange or IVIG for that indication and altered management. The patient was afebrile and hemodynamically stable at the time of the procedure, making an infectious or meningitic etiology unlikely. The cerebrospinal fluid findings summarized in Table 1 showed normal protein at 22.9 mg/dL and one nucleated cell, without albuminocytologic dissociation, arguing against Guillain-Barre syndrome and redirecting the workup toward a primary muscle disorder. The procedure was performed without complications.

This troponin elevation was interpreted as reflecting skeletal muscle injury rather than acute coronary syndrome, given the well-described cross-reactivity of high-sensitivity troponin-T assays with regenerating skeletal muscle troponin isoforms in the setting of marked rhabdomyolysis. Electrocardiography and transthoracic echocardiography, both performed to evaluate the troponin elevation, did not show evidence of acute ischemia or wall motion abnormality.

The urinalysis findings summarized in Table 1 were accompanied by moderate white blood cells and few bacteria, consistent with an asymptomatic urinary tract infection. The hepatitis C antibody was reactive, but hepatitis C virus RNA was not detected on PCR, consistent with prior cleared infection or a false-positive antibody result, and was not considered a contributor to the clinical picture. Right upper quadrant ultrasound was unremarkable, supporting a non-hepatic origin for the transaminase elevation.

Given the persistent weakness and markedly elevated CK, a standard myositis-specific antibody panel was sent. All 11 antibodies were negative on both initial and repeat testing, as summarized in Table 1. Because the standard panel was negative despite a clinical picture strongly suggestive of an autoimmune myopathy, anti-HMG-CoA reductase (anti-HMGCR) antibody was ordered as a separate, additional assay; this antibody was markedly elevated, establishing the diagnosis of anti-HMGCR antibody-positive IMNM [3]. Despite the severity of muscle injury, systemic inflammatory markers were comparatively modest, as shown in Table 1; this dissociation between high CK and modest inflammatory markers is characteristic of necrotizing myopathies.

Atorvastatin was discontinued on admission. High-dose corticosteroid therapy was initiated on hospital day 4 with prednisone 80 mg daily and a planned outpatient taper. The biochemical trajectory through hospitalization and into early outpatient follow-up is summarized in Table 2.

**Table 2:** Trajectory of creatine kinase and transaminases during hospitalization and early outpatient follow-up, with laboratory-specific normal reference ranges.

Time point	CK (U/L)	AST (U/L)	ALT (U/L)
Normal ranges	26-192	10-35	10-35
Admission (day 1)	16,237	388	407
Day 3	10,273	225	268
Day 5	9,557	201	266
Day 8	6,632	175	266
Day 15 (discharge)	5,330	163	273
Day ~30 (post-taper)	6,565	165	237

Age-appropriate malignancy screening with CT of the chest, abdomen, and pelvis was not performed during this admission. Muscle biopsy, which has historically served as the gold-standard diagnostic test for inflammatory myopathies, was not performed in this case. Under the 2018 classification criteria from the 224th European Neuromuscular Centre (ENMC) International Workshop, a diagnosis of seropositive IMNM can be established without biopsy when three criteria are met: an elevated CK, proximal muscle weakness, and a positive anti-HMGCR or anti-SRP antibody [7]. Our patient met all three criteria, which was the basis for the treating team's decision to defer biopsy; this decision and its limitations are discussed further below.

Inpatient rehabilitation and discharge

The patient was transferred to acute inpatient rehabilitation on hospital day 5 and discharged home with family support and home health services on hospital day 15. Functional gains over the 11-day rehabilitation course included progression of bed rolling, sit-to-stand, and bed-to-chair transfer from moderate assistance to independent. Ambulation, not feasible on admission for safety reasons, advanced to 150 feet with supervision and a rolling walker by discharge. Upper- and lower-body dressing, toileting hygiene, and toilet transfers all progressed to independent.

Outpatient follow-up

Outpatient electromyography (EMG) and nerve conduction studies showed abundant positive sharp waves and fibrillation potentials in proximal upper- and lower-extremity musculature, findings consistent with active inflammatory muscle disease. At neurology follow-up approximately two weeks after discharge, the prednisone dose had been tapered to 50 mg daily; within three days, CK rebounded from a post-discharge nadir of 5,330 U/L to 6,565 U/L (normal range 26-192 U/L), prompting escalation of therapy.

Neurologic examination at outpatient follow-up demonstrated severe symmetric proximal lower-extremity weakness with relative preservation of distal strength, as summarized in Table 3. Hoffmann sign, a reflex test in which flicking the nail of the middle finger downward produces involuntary flexion of the thumb and index finger when positive [8], was tested bilaterally and was absent, an expected finding given that this case involves a muscle disorder rather than an upper motor neuron or spinal cord process.

**Table 3:** Motor examination findings at outpatient neurology follow-up. Strength graded on the Medical Research Council scale (0-5, where 5 is normal full strength against resistance and 0 is no visible muscle contraction) [9].

Muscle group	Right	Left
Deltoid	3/5	4/5
Triceps, biceps, brachioradialis, wrist and finger movements	5/5	5/5
Iliopsoas	1/5	1/5
Quadriceps	1/5	1/5
Hamstrings	0/5	0/5
Hip adductors	1/5	1/5
Ankle dorsiflexion	1/5	1/5
Ankle plantarflexion	4/5	4/5

Deep tendon reflexes were diminished to absent throughout. Plantar responses were flexor (the normal, expected response when the sole of the foot is stroked). Sensory examination was intact. The patient additionally reported intermittent exertional shortness of breath and mild difficulty swallowing, raising concern for weakness of the respiratory and swallowing muscles. Intravenous immunoglobulin (IVIG) therapy was discussed in detail; informed consent was obtained after review of risks including headache, aseptic meningitis, infusion reactions, and blood clot formation; and insurance authorization was secured. IVIG was initiated at 1 gram per kilogram every four weeks (a dose on the lower end of published induction protocols, chosen given the patient's age and risk profile for blood clots) [10,11], alongside continued prednisone 50 mg daily, with planned reassessment for long-term steroid-sparing antimetabolite therapy once biochemical and functional stabilization was achieved.

Patient perspective

The patient described her symptom onset as confusing and frightening, having transitioned from one to two hours of daily ambulation and regular exercise classes to dependence on a wheelchair within weeks. She initially attributed her weakness to her semaglutide injections rather than her statin, illustrating how the absence of pain and the temporal separation between routine medications and overt symptoms can obscure the diagnostic significance of a long-tolerated drug. Her primary concerns at follow-up were the expected duration of recovery and the implications of indefinite immunosuppression.

## Discussion

This case illustrates three points relevant to the diagnosis and management of anti-HMGCR antibody-positive IMNM: the diagnostic significance of a prolonged subclinical biochemical prodrome, the necessity of dedicated anti-HMGCR testing beyond the standard myositis panel, and the role of serologic subtype in selecting second-line immunotherapy.

The subclinical biochemical prodrome

The most distinctive feature of this case is the year-long evolution of asymptomatic transaminase elevation that preceded clinical weakness, despite an additional preceding period of approximately two years of statin tolerance without laboratory abnormality. This long-tolerance-then-evolution pattern is well described in statin-induced necrotizing autoimmune myositis (SINAM), the term used in the older literature for this same disease entity: the disease can develop after months to years of previously uncomplicated statin therapy [3,12], and our patient's exposure timeline of approximately 40 months of atorvastatin before clinical presentation fits squarely within this range. The clinically actionable window is the biochemical evolution phase, during which laboratory abnormalities are detectable but symptoms have not yet developed.

AST and ALT are commonly assumed to reflect liver injury, but both enzymes are abundantly expressed in skeletal muscle, and their elevation in the absence of hepatic imaging or serologic abnormalities should prompt consideration of muscle as the source. The AHA scientific statement on statin safety notes that statin-induced liver injury typically produces ALT greater than AST, whereas muscle injury produces AST greater than ALT [2]; this guidance was developed largely in the context of acute statin-related hepatotoxicity versus myotoxicity, and its application to a slowly evolving autoimmune myopathy is less directly studied. Nevertheless, in this patient, ALT exceeded AST throughout the prodromal year, a pattern that may have falsely reassured against muscle involvement, and only shifted toward parity once the muscle-breakdown process became clinically overt. In retrospect, this trajectory almost certainly represented subclinical muscle injury evolving over months, and a single CK measurement during the prodromal period could plausibly have permitted diagnosis before the development of severe weakness.

The importance of dedicated anti-HMGCR testing

Diagnosis in this case rested entirely on isolated anti-HMGCR antibody positivity in the setting of a uniformly negative standard myositis panel. Anti-HMGCR is not included in commonly available myositis-specific antibody panels and must be requested as a separate assay [3,6]. Failure to do so in a patient with persistent weakness and elevated CK following statin discontinuation risks misclassification as antibody-negative polymyositis or unexplained muscle breakdown.

Muscle biopsy: a deferred gold standard

Muscle biopsy has historically been considered the gold-standard test for diagnosing inflammatory myopathies, since it provides direct histopathologic confirmation of muscle fiber necrosis and characterizes the pattern of inflammatory infiltrate. However, the 224th ENMC International Workshop in 2016, with criteria published in 2018, established that a diagnosis of seropositive IMNM can be made without biopsy when three criteria are satisfied: an elevated CK, proximal muscle weakness, and a positive anti-HMGCR or anti-SRP antibody [7]. Our patient met all three criteria. The decision to defer biopsy in this case carries a real tradeoff: biopsy would have provided histopathologic confirmation, additional prognostic information from the degree of muscle fiber necrosis, and would have excluded biopsy-mimicking conditions. In cases where the clinical, serologic, and biochemical findings are as concordant as they were here, many treating teams elect to forgo biopsy given its invasiveness; however, this remains a point of practice variation, and biopsy should still be strongly considered when any element of the presentation is atypical or when the response to treatment is unexpectedly poor.

Treatment: a serologic subtype-specific approach

Initial therapy for IMNM consists of high-dose glucocorticoids combined with a steroid-sparing antimetabolite (a medication such as methotrexate, azathioprine, or mycophenolate mofetil that allows the corticosteroid dose to be reduced over time), reflecting the inadequacy of steroid monotherapy and the substantial relapse rate during taper. A retrospective cohort study by Kassardjian et al. reported relapse in 16 of 29 patients (55%) during immunosuppressant taper or discontinuation across a mixed population of necrotizing autoimmune myopathy subtypes, not specifically anti-HMGCR disease [13]. Our patient's biochemical relapse within three days of tapering prednisone from 80 mg to 50 mg daily is consistent with this broader pattern. A 2026 invited review in the New England Journal of Medicine emphasizes that second-line therapy in IMNM should be selected by serologic subtype, meaning the specific autoantibody identified in a given patient. IVIG is favored as an addition to baseline corticosteroid therapy in anti-HMGCR disease, while rituximab is generally preferred in disease driven by the anti-SRP antibody [10]. A 2025 retrospective cohort study by Suh and Amato dedicated specifically to IVIG in anti-HMGCR IMNM reported CK normalization in approximately 40% of patients and improvement to normal or near-normal proximal strength in about three-quarters of patients at six months, with a measurable steroid-sparing effect [11]; together with the 2023 consensus statement from the American Association of Neuromuscular and Electrodiagnostic Medicine (AANEM) endorsing IVIG as a second-line agent in this setting [14], these data support the selection of IVIG over rituximab in our patient. The smaller retrospective French series of rituximab in refractory anti-HMGCR disease reported response in only one-third of patients [15], reinforcing the rationale for IVIG-first escalation when anti-HMGCR is the defining serology.

In our patient, IVIG was initiated at 1 gram per kilogram every four weeks (a dose on the lower end of published induction protocols, chosen given the patient's age and risk profile for blood clots) [10,11], alongside continued prednisone 50 mg daily. This sequencing defers antimetabolite addition pending assessment of IVIG tolerance and response, with the understanding that combination therapy would be revisited at the next follow-up if stabilization was incomplete.

Glucocorticoid-induced osteoporosis

An additional consideration in this case is the risk of glucocorticoid-induced bone loss. The patient received prednisone at doses of 80 mg daily, tapering to 50 mg daily, over a period of at least several weeks. The American College of Rheumatology guideline for prevention and treatment of glucocorticoid-induced osteoporosis recommends oral bisphosphonate therapy for adults 30 years of age or older who receive a very high-dose glucocorticoid course, defined as an initial prednisone dose of 30 mg per day or more, or a cumulative annual dose exceeding 5 grams; this patient's regimen meets that threshold [16]. Review of the available medical records did not identify documentation of calcium and vitamin D supplementation, bone mineral density assessment, or bisphosphonate therapy at any point during the hospitalization or early outpatient follow-up. This represents a missed opportunity for bone-protective intervention and is addressed further in the Limitations section below.

Long-term considerations

Statin rechallenge is contraindicated, and lifelong statin avoidance is the standard recommendation [17]. Alternative lipid-lowering agents that act independently of skeletal muscle HMG-CoA reductase (including ezetimibe, PCSK9 inhibitors, and bempedoic acid) have been used safely in anti-HMGCR antibody-positive patients and will require longitudinal coordination with primary care. Given the recognized association between IMNM and occult malignancy in patients over 50, with a reported 2.8- to 9.1-fold increased risk [12], we recommend that this patient complete age-appropriate malignancy screening with CT of the chest, abdomen, and pelvis on an outpatient basis, since this was not performed during the index admission.

Limitations

Several limitations of this case warrant acknowledgment. Muscle biopsy, the historical gold standard for diagnosing inflammatory myopathies, was not performed, for the reasons discussed above. Dedicated MRI of the affected proximal lower-extremity musculature was not obtained, which would have provided supportive radiographic evidence and a quantitative baseline for treatment monitoring. Dedicated cardiac MRI was not obtained despite the critical high-sensitivity troponin-T elevation. Malignancy screening with CT of the chest, abdomen, and pelvis was not performed during the index admission. Despite a prednisone course meeting the threshold for very high-dose glucocorticoid exposure, calcium and vitamin D supplementation, bone mineral density assessment, and bisphosphonate therapy do not appear to have been addressed, representing a missed opportunity for fracture-prevention intervention that should be prioritized at subsequent outpatient visits. Finally, follow-up extends only through initial IVIG planning; longer-term outcomes including strength recovery, steroid wean, and durability of response remain to be determined.

## Conclusions

Anti-HMGCR antibody-positive IMNM is an underdiagnosed cause of progressive proximal weakness in statin-treated patients, and its prodrome may unfold over many months as asymptomatic transaminase elevation that is readily misattributed to liver injury. Measuring CK alongside transaminases in any statin-treated patient with unexplained liver enzyme elevation represents a simple, actionable step toward earlier diagnosis. When IMNM is suspected, anti-HMGCR antibody testing must be requested separately from the standard myositis panel. Second-line immunotherapy should be guided by serologic subtype, with current evidence supporting IVIG as the preferred adjunct in anti-HMGCR disease.
